# Assessment of Osteoporosis in Lumbar Spine: *In Vivo* Quantitative MR Imaging of Collagen Bound Water in Trabecular Bone

**DOI:** 10.3389/fendo.2022.801930

**Published:** 2022-02-16

**Authors:** Jin Liu, Jian-Wei Liao, Wei Li, Xiao-Jun Chen, Jia-Xin Feng, Lin Yao, Pan-Hui Huang, Zhi-Hai Su, Hai Lu, Yu-Ting Liao, Shao-Lin Li, Ya-Jun Ma

**Affiliations:** ^1^Department of Radiology, The Fifth Affiliated Hospital of Sun Yat-Sen University, Zhuhai, China; ^2^Department of Spinal Surgery, The Fifth Affiliated Hospital of Sun Yat-Sen University, Zhuhai, China; ^3^MR Research, GE Healthcare, Guangzhou, China; ^4^Department of Radiology, University of California San Diego, La Jolla, CA, United States

**Keywords:** osteoporosis, ultrashort echo time, collagen bound water proton density, bone mineral density, bone marrow fat fraction

## Abstract

**Aim:**

Bone collagen matrix makes a crucial contribution to the mechanical properties of bone by imparting tensile strength and elasticity. The collagen content of bone is accessible *via* quantification of collagen bound water (CBW) indirectly. We prospectively study the performance of the CBW proton density (CBWPD) measured by a 3D short repetition time adiabatic inversion recovery prepared ultrashort echo time (STAIR-UTE) magnetic resonance imaging (MRI) sequence in the diagnosis of osteoporosis in human lumbar spine.

**Methods:**

A total of 189 participants with a mean age of 56 (ranged from 50 to 86) years old were underwent MRI, quantitative computed tomography (QCT), and dual-energy X-ray absorptiometry (DXA) in lumbar spine. Major fracture risk was also evaluated for all participants using Fracture Risk Assessment Tool (FRAX). Lumbar CBWPD, bone marrow fat fraction (BMFF), bone mineral density (BMD) and T score values were calculated in three vertebrae (L2–L4) for each subject. Both the CBWPD and BMFF were correlated with BMD, T score, and FRAX score for comparison. The abilities of the CBWPD and BMFF to discriminate between three different cohorts, which included normal subjects, patients with osteopenia, and patients with osteoporosis, were also evaluated and compared using receiver operator characteristic (ROC) analysis.

**Results:**

The CBWPD showed strong correlation with standard BMD (R^2^ = 0.75, P < 0.001) and T score (R^2^ = 0.59, P < 0.001), as well as a moderate correlation with FRAX score (R^2^ = 0.48, P < 0.001). High area under the curve (AUC) values (≥ 0.84 using QCT as reference; ≥ 0.76 using DXA as reference) obtained from ROC analysis demonstrated that the CBWPD was capable of well differentiating between the three different subject cohorts. Moreover, the CBWPD had better correlations with BMD, T score, and FRAX score than BMFF, and also performed better in cohort discrimination.

**Conclusion:**

The STAIR-UTE-measured CBWPD is a promising biomarker in the assessment of bone quality and fracture risk.

## Introduction

Osteoporosis (OP) has become a major public health burden as the population continues to age (1, 2). Each year, about 200 million people worldwide suffer from OP, with approximately 89 million fractures occurring on an annual basis (3). OP is characterized by a decrease in bone strength combined with an increased risk of fracture as a result of low bone mass and microarchitectural deterioration of bone tissue (4).

Bone mineral density (BMD) plays a major role in bone strength and its measurement of trabecular bone in the spine and/or hip using dual-energy x-ray absorptiometry (DXA) is the clinical standard for assessments of both OP and fracture risk (5–7). Quantitative computed tomography (QCT) utilizes 3D volumetric imaging and quantification to further improve the accuracy of these BMD measurements but with increased ionizing radiation.

The organic matrix, another important bone component, provides tensile strength and elasticity, also contributing significantly to the mechanical properties of bone (8). Because changes in the collagen density and integrity within the bone’s organic matrix affect overall bone quality (9), quantitative evaluation of the collagen matrix is likely to provide valuable information on an individual’s bone strength (10–12).

Unfortunately, both DXA and QCT are limited in their abilities to assess changes in the collagen matrix due to their low soft tissue contrast. Magnetic resonance imaging (MRI) is a superior method in that it offers high-contrast soft tissue imaging. However, collagen cannot be directly imaged by MRI due to its extremely short T_2_ relaxation time (about 10 µs) (13–15). Alternatively, indirect evaluation of the collagen matrix can be performed by quantification of the water molecules which are tightly bound to the collagen matrix (i.e., collagen bound water (CBW)) and are highly correlated with the matrix collagen’s density and integrity (16). Like collagen itself, the CBW also has a relatively short T_2_ relaxation time—around 300 µs. While CBW is not accessible to clinical sequences with long echo times (TEs) (i.e., several to hundreds of milliseconds) such as gradient recalled echo (GRE) and fast spin echo (FSE) (17, 18), a specialized MRI technique known as the ultrashort echo time (UTE) sequence has been developed with a TE less than 100 µs to specifically target tissues with short T_2_ relaxation times. This UTE MRI approach can be used to image and quantify the CBW (19).

In the last decade, UTE MRI imaging of CBW has been studied by several groups as a possible surrogate measure for the bone’s collagen matrix (18, 20–23). It was found that the UTE measurements scale to an almost linear degree with collagen matrix density (22, 24, 25), and highly correlated with yield, peak stress, and elastic toughness (25, 26). However, most of the previous studies were focused on CBW measurement in cortical bone. CBW evaluation in trabecular bone has the potential to be even more valuable a measurement because not only do most osteoporotic fractures occur in trabecular bone, but trabecular bone is also highly responsive to metabolic stimuli (27–30). However, quantification of CBW in trabecular bone is more technically challenging than in cortical bone due to trabecular bone’s much lower CBW content and higher concentration of tissue components with long T_2_ relaxation times, such as marrow fat (31).

Most recently, a new 3D short repetition time adiabatic inversion recovery prepared UTE (3D STAIR-UTE) Cones sequence was developed which could volumetrically quantify CBW proton density (CBWPD) of trabecular bone *in vivo* for the first time (17). The STAIR-UTE sequence can generate high contrast imaging of CBW with sufficient suppression of all the long T_2_ signals.

The purpose of our study was to investigate the STAIR-UTE-measured CBWPD in the classification of patients as normal, osteopenic and osteoporotic in the lumbar spine with golden standard QCT-measured BMD and DXA-measured T score as reference. The CBWPD was also correlated with fracture risk score which was characterized by Fracture Risk Assessment Tool (FRAX). Moreover, vertebral bone marrow fat fraction (BMFF)—another quantitative imaging biomarker that has been studied extensively in OP assessment—was also employed for comparison against CBWPD.

## Materials and Methods

### Subject Recruitment

This study was conducted under Institutional Review Board approval; written informed consent was obtained from all participants. Between June 2020 and January 2021, 189 participants with a mean age of 56 (ranged from 50 to 86) years old were recruited to participate in this prospective study. MRI, QCT, and DXA examinations of the lumbar spine for each participant were performed in the same week. Subjects were excluded if they had known preexisting bone diseases (e.g., lumbar fracture, tumor, metastases, dysplasia, or metabolic disorders), a history of lumbar surgery, or a history of drug therapy targeted at BMD. We also excluded any subjects who had a history of osteoarthritis, inflammatory arthritis, cancer, Paget disease, endocrinologic or gastrointestinal disorder, glucocorticoid use, selective serotonin uptake inhibitor use, or anticonvulsant use.

### QCT and DXA Examinations

QCT examinations of the lumbar region were performed on a 128-channel multi-detector CT scanner (uCT 760, United Imaging Healthcare). All CT parameters were set in accordance with the “China Health Quantitative CT Big Data Project Research Program” (32) as follows: collimation: 0.625 mm, tube voltage: 120 kVp, tube current: automatic. All CT images were reconstructed to 512×512 matrices using iterative reconstruction algorithms available from the vendor’s CT scanners. The reconstruction intervals were 1.0 mm. Our QCT post-processing can only quantify three vertebral bodies at a time, so the center three vertebral elements of the lumbar spine (i.e., L2-L4) were chosen for the quantification.

Standard DXA scan (Osteocore, Medilink 90kv and 2mA) was performed according to WHO guidelines (33) to acquire T score in the lumbar spine. Images of the lumbar spine were obtained in posterior-anterior projection.

### MR Imaging

All 189 participants underwent spine MR imaging on a 3T clinical scanner (Pioneer, GE Healthcare Technologies, Milwaukee, WI) with a spine-array surface coil for signal reception. Clinical sagittal T_1_-weighted FSE (repetition time (TR): 540 ms, TE: 8 ms, flip angle (FA): 80°, field-of-view (FOV): 32×32 cm^2^, pixel size: 1.0×1.4 mm^2^, number of slices: 13, slice thickness: 4 mm, scan time: 1 min) and T_2_-weighted FSE (TR: 2291 ms, TE: 90 ms, FA: 90°, FOV: 32×32 cm^2^, pixel size: 1.0×1.4 mm^2^, number of slices: 13, slice thickness: 4 mm, scan time: 2 min) scans of the lumbar spine were included. A product sequence, IDEAL-IQ (Iterative decomposition of water and fat with echo asymmetry and least squares estimation) (34), was used to quantify BMFF (TR: 7.3 ms; TEs: 1.2, 2.1, 3.1, 4.1, 5.0, and 6.0 ms; FA: 4°; FOV: 32×32 cm^2^; pixel size: 2.0×2.0 mm^2^; number of slices: 12; slice thickness: 8 mm; and scan time: 16 sec).

The 3D STAIR-UTE Cones sequence was performed in the sagittal plane for the CBW imaging (17) (TR: 150 ms, TE: 0.032 ms, inversion time (TI): 64 ms, FA: 18°, number of spokes per-TR: 5, spoke interval: 5.5 ms, FOV: 30 cm×30 cm, pixel size: 2.1×2.1mm^2^, number of slices: 16, slice thickness: 4.5 mm, oversampling factor: 2, scan time: 10 min). In order to acquire a spine coil sensitivity profile for correction of the STAIR-UTE imaging inhomogeneity, the 3D UTE Cones sequence was applied twice without STAIR preparation, using spine and body coils for signal reception, respectively (17) (TR: 6 ms, TE: 0.032 ms, FA: 2°, FOV: 30 cm×30 cm; pixel size, 2.1×2.1 mm^2^, number of slices: 16, slice thickness: 4.5 mm, and scan time: 1 min. A rubber band with a T2* of 0.34 ms and a premeasured proton density of 18 mol/L was placed between the spine coil and participants during scanning to serve as a reference standard. The CBWPD of trabecular bone was calculated using Equation [9] in Ref (17).. Ten healthy volunteers were recruited to study the reproducibility of the STAIR-UTE Cones sequence. Each volunteer was scanned three times over three consecutive days.

### Fracture Risk Characterization

The major fracture risk (i.e., 10-year probability of osteoporotic fracture) of each participant was characterized using FRAX (https://www.sheffield.ac.uk/FRAX) with 11 clinical variables taken into consideration (i.e., age, sex, weight, height, previous fracture, parental hip fracture, current smoking status, glucocorticoid use, rheumatoid arthritis, secondary OP, and alcohol use of three or more units per day).

### Data Analysis

The CBWPD calculation was performed in MATLAB (The MathWorks, Natick, MA). Regions of interest (ROIs) were manually drawn in the trabecular bone region—avoiding the subchondral bone region—to measure BMD, CBWPD, and BMFF in L2–L4. Mean lumbar BMD, CBWPD, and BMFF values were computed by averaging them across these three vertebrae for each subject. ROIs were independently drawn by two radiologists with 10 (W.L.) and 8 (J.L.) years of respective experience to assess inter-observer agreement of ROIs. To assess intra-observer agreement of ROI drawing, this procedure was repeated by the radiologist with 10 years of experience two months after the initial ROI drawing.

For the DXA experiment, T score was used to assess osteoporosis since all the participants were over 50 years old according to WHO guidelines (35). The scanned subjects were grouped into three cohorts (normal, osteopenia, and OP). A BMD (measured by QCT) greater than 120 mg/cm^3^ (equivalent to a DXA T score > -1.0 standard deviation (SD)) indicates normal, a BMD value between 80 mg/cm^3^ and 120 mg/cm^3^ (equivalent to a DXA T score < -1.0 SD and > -2.5 SD) indicates osteopenia, and a BMD less than 80 mg/cm^3^ (equivalent to a DXA T score of < -2.5 SD) indicates OP.

### Statistical Analysis

Statistical analysis was performed using Statistical Package for Social Sciences (SPSS) software (version 23.0). P < 0.05 was considered statistically significant. Linear regression and Bland–Altman analysis were performed to assess the reproducibility of the STAIR-UTE Cones sequence. Intra- and interclass correlation coefficients (ICC) were calculated to assess both the interobserver and intra-observer reproducibility of CBWPD and BMFF measurements. The differences in age, body mass index (BMI), BMD, T score, CBWPD, BMFF, and FRAX score among all three cohorts (normal, osteopenia, and OP) were determined using the Kruskal-Wallis test. The differences in sex among all three cohorts were determined using the Chi-square test. Linear regression was performed to correlate the CBWPD and BMFF with BMD and T score, respectively. Non-linear regression using an exponential function was performed to correlate the CBWPD and BMFF with FRAX score. To evaluate the performances of the CBWPD and BMFF in discriminating between the three cohorts, receiver operating characteristic (ROC) analysis was performed and the area under the curve (AUC) with 95% confidence interval (CI) was computed using Medcalc software (version 20.0.3). The DeLong test was used to compare the ROC curves (i.e., AUCs) between CBWPD and BMFF.

## Results

Data analysis was performed on 162 of the recruited 189 participants, with the 27 exclusions made due to bone disease (n = 17) and poor MR image quality caused by motion during the scan (n = 10). Participant characteristics are described in **Table 1**. Age, BMD, T score, CBWPD, BMFF, and FRAX score all showed significant differences among the three cohorts, with QCT and DXA used as the respective reference standards.

**Table 1:** Characteristics of patients in three cohorts of normal subjects, patients with osteopenia, and patients with osteoporosis.

		QCT as reference standard				DXA as reference standard			
	All subjects (n=162)	Normal(n = 86)	Osteopenia (n = 41)	Osteoporosis (n = 35)	*P*Value	Normal (n = 88)	Osteopenia (n = 46)	Osteoporosis (n = 28)	*P*Value
Sex					0.198				0.148
male	65 (40%)	35 (41%)	20 (49%)	10 (29%)		36 (41%)	22 (48%)	7 (25%)	
female	97 (60%)	51 (59%)	21 (51%)	25 (71%)		52 (59%)	24 (52%)	21 (75%)	
Age (years)	58 (9)	53 (5)	60 (8)	68 (9)	<0.001	55 (6)	60 (9)	66 (10)	<0.001
BMI (kg/m^2^)	23.4 (21.3-25.4)	23.4 (21.5-25.5)	23.8 (21.1-26.8)	22.9 (20.5-25.0)	0.517	23.7 (21.7-26.0)	23.9 (21.3-26.1)	21.68 (20.1-23.6)	0.004
BMD (mg/cm^3^)	119.8 (83.6-154.7)	155.6 (132.5-175.7)	100.7 (90.0-111.1)	54.1 (43.9-67.9)	<0.001	148.9 (125.5-174.7)	99.8 (80.2-120.6)	60.9 (47.9-71.1)	<0.001
T score	0.5 (-2.1 to -0.6)	0.8 (-0.3 to 2.0)	-1.3 (-2.0 to -0.3)	-2.7 (-3.4 to -2.3)	<0.001	1.0 (0.0 to 2.0)	-1.7 (-2.1 to -1.3)	-3.1 (-3.5 to -2.6)	<0.001
CBWPD (mol/L)	2.5 (1.9-2.9)	2.9 (2.5-3.3)	2.3 (2.1-2.5)	1.6 (1.3-1.8)	<0.001	2.9 (2.4-3.2)	2.2 (1.8-2.4)	1.7 (1.4-1.9)	<0.001
BMFF (%)	54.2 (44.4-63.3)	48.0 (41.2-55.2)	55.8 (48.2-62.8)	67.5 (60.0-73.4)	<0.001	49.2 (42.0-56.5)	57.1 (46.7-67.9)	65.0 (58.1-72.7)	<0.001
FRAX score (%)	3.7 (1.5-4.7)	1.9 (1.2-2.0)	3.80 (1.9-5.0)	7.8 (4.6-9.5)	<0.001	1.7 (1.2-1.9)	3.9 (2.3-4.8)	9.3 (6.2-10.0)	<0.001

Data are presented as n (%), mean (SD), or median (IQR). All comparisons between the three cohorts were significant (P < 0.001) except for sex (P = 0.198, taking QCT as reference standard; P = 0.148, taking DXA as reference standard) and BMI (P = 0.517, taking QCT as reference standard; P = 0.004, taking DXA as reference standard).

BMI, body mass index; CBWPD, collagen bound water proton density; QCT, quantitative computed tomography; BMD, bone mineral density; DXA, dual-energy X-ray absorptiometry; BMFF, bone marrow fat fraction; FRAX, Fracture Risk Assessment Tool; IQR, interquartile range.

**Figure 1:**
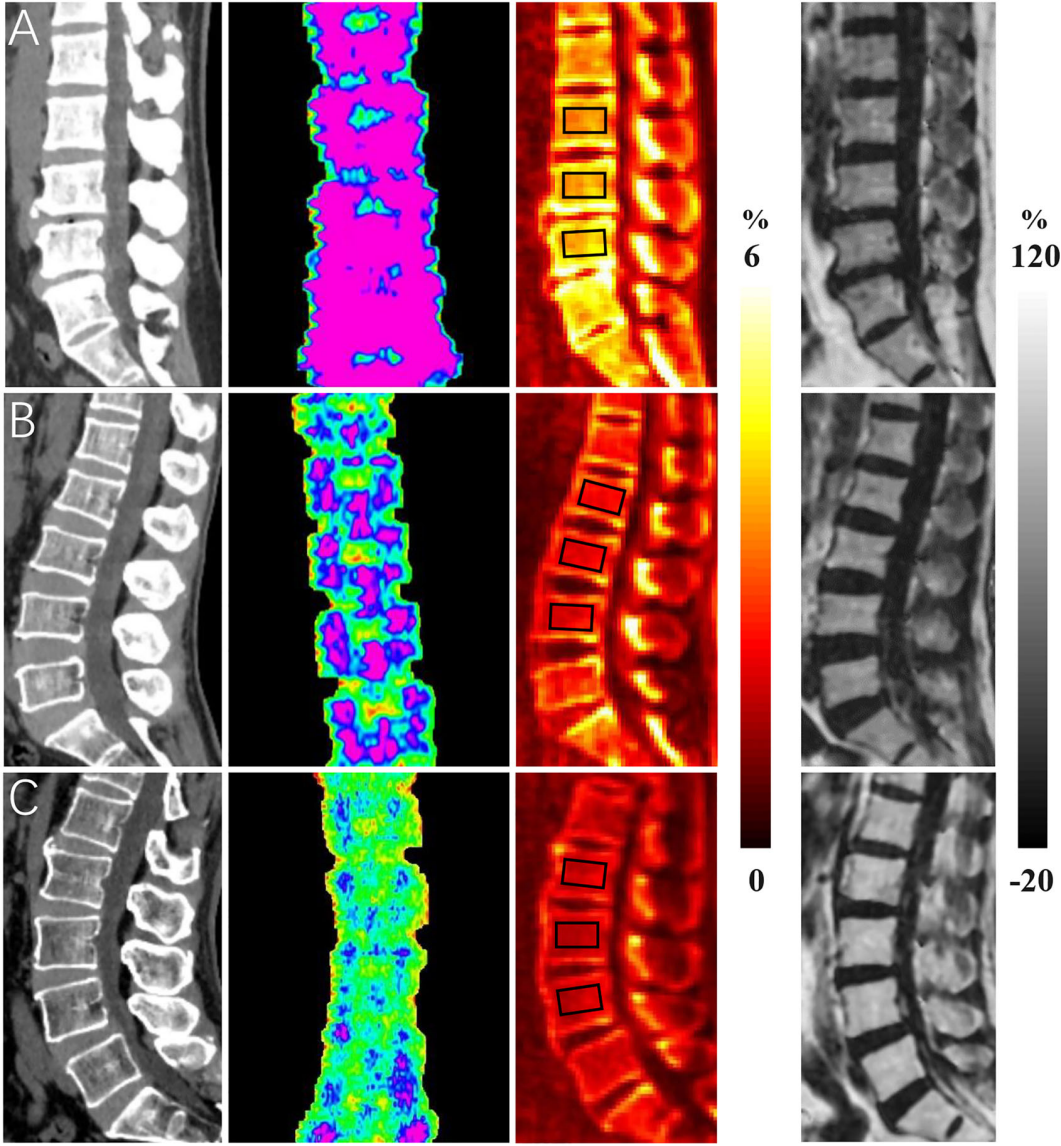
Representative bone mineral density (BMD) (first column), T score (second column), collagen bound water proton density (CBWPD) (third column), and bone marrow fat fraction (BMFF) (fourth column) maps in the lumbar spine of three subjects with normal bone mass (first row **(A)**, 50-year-old male), osteopenia (second row **(B)**, 54-year-old female), and osteoporosis (last row **(C)**, 66-year-old male). ROIs inside of black squares were drawn for data analysis.

**Figure 1** shows representative BMD, T score, CBWPD, and BMFF maps of the lumbar spines of three subjects: one from each cohort (normal, osteopenia, and OP). Lower CBWPD, BMD, and T score, as well as higher BMFF, were found in more osteoporotic subjects.

### Reproducibility

**Figure 2:**
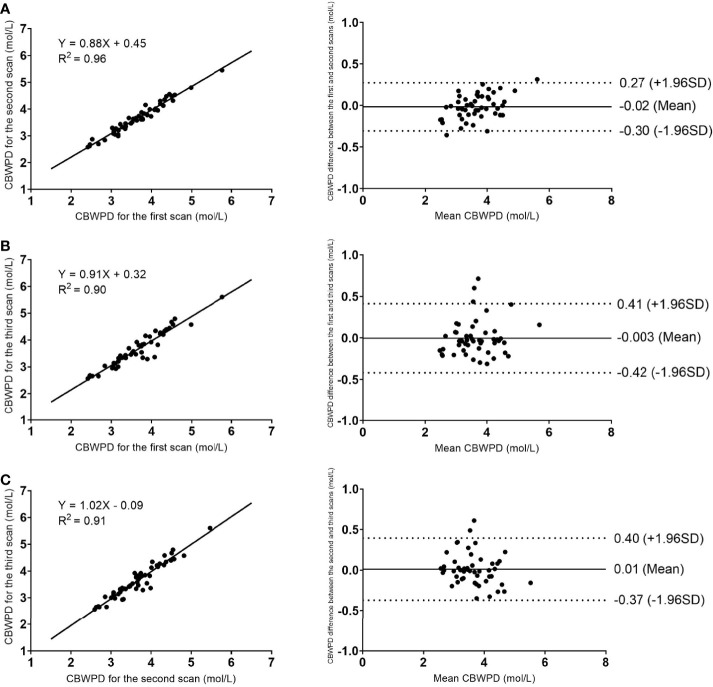
Reproducibility results of the STAIR-UTE Cones scans: linear regression (left column) and Bland–Altman analysis (right column) plots of the STAIR-UTE measured CBWPDs between the first and the second scans **(A)**, between the first and the third scans **(B)**, and between the second and the third scans **(C)**. Dotted lines in the Bland–Altman difference plots (right column) demarcate 1.96 standard deviations of the mean difference.

The results of linear regression and Bland–Altman analysis of the STAIR-UTE measured CBWPDs for the three repeated volunteer scans are shown in **Figure 2**. Strong correlations and high agreements of the CBWPD measurements were found between the first and the second scans with a R^2^ = 0.96 (P < 0.001) and a mean bias of -0.02 mol/L (± 1.96 SD: range –0.30 to 0.27 mol/L) (**Figure 2A**) respectively, between the first and the third scans with a R^2^ = 0.90 (P < 0.001) and a mean bias of -0.003 mol/L (± 1.96 SD: range –0.42 to 0.41 mol/L) (**Figure 2B**) respectively, and between the second and the third scans with a R^2^ = 0.91 (P < 0.001) and a mean bias of 0.01 mol/L (± 1.96 SD: range –0.37 to 0.40 mol/L) (**Figure 2C**) respectively. These results demonstrate an excellent reproducibility for the STAIR-UTE Cones scans.

The interobserver ICCs for the CBWPD and BMFF measurements between the two radiologists were 0.93 and 0.92, respectively. The intra-observer ICCs for the CBWPD and BMFF quantification were 0.95 and 0.93, respectively. These high ICC values demonstrate excellent reproducibility for both interobserver and intra-observer measurements.

### Correlations

**Figure 3:**
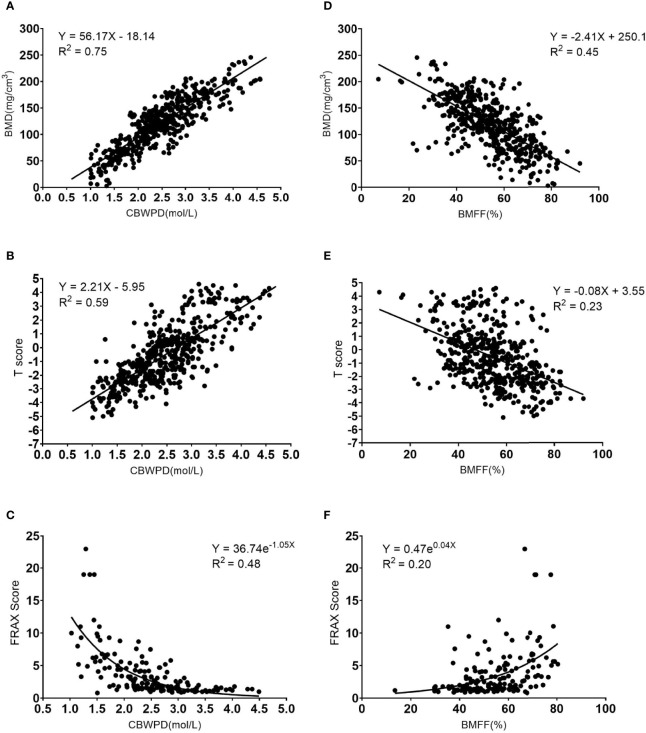
Correlation curves and scatter plots for the measurements between **(A)** collagen bound water proton density (CBWPD) and bone mineral density (BMD), **(B)** CBWPD and T score, **(C)** CBWPD and Fracture Risk Assessment Tool (FRAX) score, **(D)** bone marrow fat fraction (BMFF) and BMD, **(E)** BMFF and T score, and **(F)** BMFF and FRAX score.

The CBWPD showed a positive correlation with BMD (R^2^ = 0.75, P < 0.001) (**Figure 3A**), a positive correlation with T score (R^2^ = 0.59, P < 0.001) (**Figure 3B**), and a negative correlation with FRAX score (R^2^ = 0.48, P < 0.001) (**Figure 3C**).

The BMFF showed a negative correlation with BMD (R^2^ = 0.45, P < 0.001) (**Figure 3D**), a negative correlation with T score (R^2^ = 0.23, P < 0.001) (**Figure 3E**), and a positive correlation with FRAX score (R^2^ = 0.20, P < 0.001) (**Figure 3F**).

As demonstrated by these results, the CBWPD correlates better with BMD, T score, and FRAX score than the BMFF does.

### ROC Analysis

**Figure 4:**
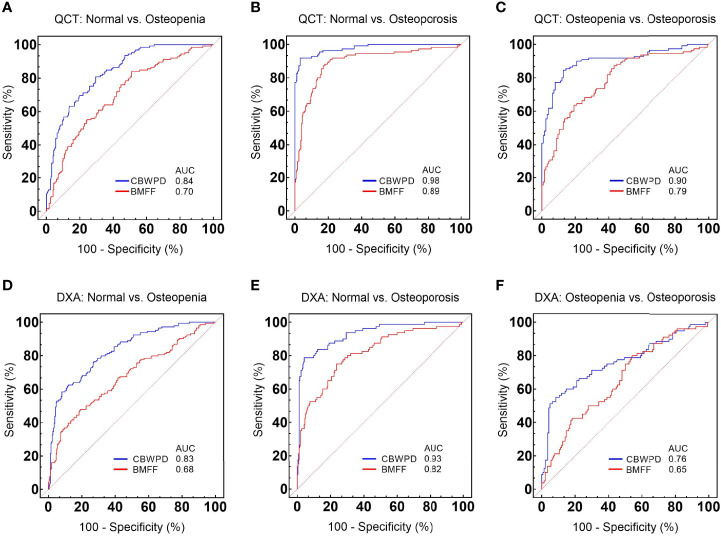
Receiver operating characteristic (ROC) curves and corresponding area under the curve (AUC) values of collagen bound water proton density (CBWPD^QCT^) and bone marrow fat fraction (BMFF^QCT^) between **(A)** normal and osteoporosis, **(B)** normal and osteopenia, and **(C)** osteopenia and osteoporosis, with quantitative computed tomography (QCT) as reference standard. ROC curves and corresponding AUC values of CBWPD^DXA^ and BMFF^DXA^ between **(D)** normal and osteoporosis, **(E)** normal and osteopenia, and **(F)** osteopenia and osteoporosis, with dual-energy X-ray absorptiometry (DXA) as reference standard.

**Figures 4A–C** show the ROC curves of CBWPD^QCT^ and BMFF^QCT^ (with QCT as reference standard) in differentiating normal subjects, patients with osteopenia, and patients with OP. **Table 2** summarizes all the criteria that were used to evaluate the diagnostic performance of CBWPD^QCT^ and BMFF^QCT^. The AUC values of CBWPD^QCT^ and BMFF^QCT^ were 0.84 and 0.70 (P < 0.001) for normal vs. osteopenia, 0.98 and 0.89 (P < 0.001) for normal vs. OP, and 0.90 and 0.79 (P < 0.001) for osteopenia vs. OP, respectively. The AUC values of CBWPD^QCT^ were consistently higher than the corresponding AUC values of BMFF^QCT^.

**Table 2:** Performance of the CBWPD^QCT^ and BMFF^QCT^ in discrimination between three cohorts of normal subjects, patients with osteopenia, and patients with osteoporosis, with QCT as reference standard.

	CBWPD^QCT^			BMFF^QCT^			
	Normal vs. osteopenia	Normal vs. osteoporosis	Osteopenia vs. osteoporosis	Normal vs. osteopenia	Normal vs. osteoporosis	Osteopenia vs. osteoporosis
AUC (95%CI)	0.839 (0.799, 0.879)	0.978 (0.963, 0.992)	0.902 (0.861, 0.944)	0.704 (0.649, 0.759)	0.894 (0.854, 0.933)	0.786 (0.728, 0.845)
Sensitivity (95%CI)	0.705 (0.649, 0.762)	0.968 (0.946, 0.990)	0.872 (0.813, 0.931)	0.840 (0.776, 0.904)	0.882 (0.821, 0.942)	0.636 (0.546, 0.726)
Specificity (95%CI)	0.808 (0.739, 0.877)	0.918 (0.867, 0.969)	0.845 (0.778, 0.913)	0.490 (0.428, 0.552)	0.821 (0.773, 0.868)	0.808 (0.739, 0.877)
ACC (95%CI)	0.739 (0.738, 0.740)	0.953 (0.953, 0.953)	0.860 (0.859, 0.861)	0.606 (0.605, 0.608)	0.839 (0.839, 0.840)	0.728 (0.726, 0.729)
PPV (95%CI)	0.881 (0.836, 0.925)	0.964 (0.941, 0.987)	0.865 (0.805, 0.925)	0.451 (0.387, 0.515)	0.683 (0.607, 0.760)	0.745 (0.657, 0.833)
NPV (95%CI)	0.577 (0.504, 0.650)	0.927 (0.878, 0.976)	0.853 (0.787, 0.920)	0.860 (0.803, 0.917)	0.941 (0.909, 0.972)	0.716 (0.642, 0.791)

CBWPD, collagen bound water proton density; BMFF, bone marrow fat fraction; QCT, quantitative computed tomography; AUC, area under curve; ACC, Accuracy; PPV, Positive Predictive Value; NPV, Negative Predictive Value; CI, Confidence Interval.

**Figures 4D–F** show the ROC curves of CBWPD^DXA^ and BMFF^DXA^ (with DXA as reference standard) in differentiating normal subjects, patients with osteopenia, and patients with OP. **Table 3** summarizes all the criteria that were used to evaluate the diagnostic performance of CBWPD^DXA^ and BMFF^DXA^. The AUC values of CBWPD^DXA^ and BMFF^DXA^ were 0.83 and 0.68 (P < 0.001) for normal vs. osteopenia, 0.93 and 0.82 (P < 0.001) for normal vs. OP, and 0.76 and 0.65 (P = 0.005) for osteopenia vs. OP, respectively. The AUC values of CBWPD^DXA^ were consistently higher than the corresponding AUC values of BMFF^DXA^.

**Table 3:** Performance of the CBWPD^DXA^ and BMFF^DXA^ in discrimination between three cohorts of normal subjects, patients with osteopenia, and patients with osteoporosis, with DXA as reference standard.

	CBWPD^DXA^			BMFF^DXA^		
	Normal vs. osteopenia	Normal vs. osteoporosis	Osteopenia vs. osteoporosis	Normal vs. osteopenia	Normal vs. osteoporosis	Osteopenia vs. osteoporosis
AUC (95%CI)	0.832 (0.792, 0.873)	0.930 (0.897, 0.963)	0.761 (0.690, 0.831)	0.676 (0.619, 0.732)	0.816 (0.760, 0.872)	0.655 (0.580, 0.729)
Sensitivity (95%CI)	0.882 (0.843, 0.921)	0.958 (0.934, 0.982)	0.917 (0.872, 0.962)	0.479 (0.398, 0.561)	0.750 (0.655, 0.845)	0.800 (0.712, 0.888)
Specificity (95%CI)	0.625 (0.546, 0.704)	0.788 (0.698, 0.877)	0.550 (0.441, 0.659)	0.809 (0.762, 0.857)	0.763 (0.712, 0.815)	0.458 (0.377, 0.540)
ACC (95%CI)	0.791 (0.790, 0.791)	0.918 (0.918, 0.919)	0.786 (0.784, 0.787)	0.692 (0.691, 0.693)	0.760 (0.759, 0.761)	0.580 (0.578, 0.582)
PPV (95%CI)	0.811 (0.765, 0.856)	0.937 (0.907, 0.966)	0.786 (0.724, 0.848)	0.580 (0.491, 0.669)	0.492 (0.403, 0.581)	0.451 (0.369, 0.533)
NPV (95%CI)	0.744 (0.666, 0.822)	0.851 (0.770, 0.932)	0.786 (0.678, 0.893)	0.739 (0.688, 0.790)	0.909 (0.871, 0.947)	0.805 (0.719, 0.891)

CBWPD, collagen bound water proton density; BMFF, bone marrow fat fraction; DXA, dual-energy X-ray absorptiometry; AUC, area under curve; ACC, Accuracy; PPV, Positive Predictive Value; NPV, Negative Predictive Value; CI, Confidence Interval.

As demonstrated by these results, the CBWPD shows high capability to classify patients as normal, osteopenic and osteoporotic. It also performs better than the BMFF in discrimination of the three cohorts whether using QCT or DXA as the reference standard.

## Discussion

In this prospective study, we presented a noninvasive and nondestructive MRI technique to measure CBWPD in the human lumbar spine for assessment of trabecular bone quality using QCT and DXA as the reference standard. To the best of our knowledge, this is the first study of trabecular CBWPD in the diagnosis of OP *in vivo*. The CBWPD showed strong correlations with standard BMD (R^2^ = 0.75) and T score (R^2^ = 0.59), as well as a moderate correlation with FRAX score (R^2^ = 0.48). High AUC values (≥ 0.84 using QCT as reference, ≥ 0.76 using DXA as reference) obtained from ROC analysis demonstrated that the CBWPD was capable of discriminating between the three subject cohorts which included normal subjects, patients with osteopenia, and patients with OP. Moreover, the CBWPD had stronger correlations with BMD, T score, and FRAX score than BMFF did, and also performed better in cohort discrimination. This study demonstrates that the STAIR-UTE-measured CBWPD is a promising biomarker to be used in clinical practice for assessment of bone quality and fracture risk.

The STAIR-UTE sequence used in this study is able to efficiently suppress all the long T_2_ components in trabecular bone, including marrow fat and free water, and selectively image CBW. As can be seen in Ref. 17, numerical simulation results showed that, when a sufficiently short TR was used, the STAIR-UTE sequence achieved efficient signal suppression for long T_2_ tissues with a wide range of T_1s_. When a minimal TR (i.e., TR = 150 ms, restricted by SAR limitation) was used in the STAIR-UTE sequence on a clinical 3T scanner, only short T_2_ signals were detected in the *in vivo* vertebral bone with a T_2_* of 0.31 ms, clearly demonstrating the technical feasibility of the STAIR-UTE sequence in sufficient suppression of all the long T_2_ signals. At the long T_2_ signal null point (TI_null_), signal from the short T_2_ component (i.e., the CBW) can be efficiently detected by the 3D UTE Cones sequence with a minimal TE of 32 µs. In addition, the relatively low STAIR-UTE resolution used in this study was able to increase the SNR of short T_2_ imaging and thereby improve the accuracy of CBW quantification.

Approximately 40-50% of women and 13-22% of men are at risk of osteoporotic fracture after the age of 50, and major osteoporotic fractures result in substantial morbidity and mortality (2, 36). Early assessment of bone quality and fracture risk is crucial for effective intervention or treatment. The collagen matrix makes a crucial contribution to the mechanical properties of bone by imparting tensile strength and elasticity (37). However, changes in the organic matrix are inaccessible to DXA, QCT, and even clinical MRI due to the technical limitations of these imaging modalities (14, 15).

The STAIR-UTE MRI technique is capable of indirectly accessing properties in the collagen matrix by performing measurements of the CBWPD in trabecular bone (17). The CBW provides important information regarding the collagen density and hydration state of the organic matrix to which it is bound. In this way, CBW quantification could act as a possible surrogate for assessment of the collagen matrix.

It is known that mineral crystals are scattered in the gaps between continuous collagen fibers (38), suggesting that the quantity of collagen fibers may be related to the BMD. In our trabecular bone study, the strong positive correlation between CBWPD and BMD was in line with the findings in previous cortical bone studies as it demonstrated that both the bone mineral and organic matrix were decreased in OP (39–41).

A moderate correlation between the CBWPD and FRAX score demonstrated that the STAIR-UTE-measured CBWPD has the potential to be useful in assessing fracture risk or could even be combined into the FRAX score calculation for further improved risk assessment. Another advantage of the STAIR-UTE MRI technique is the absence of ionizing radiation which may make it a preferable technique for regular examinations (an important approach for early detection of bone loss) or longitudinal studies that monitor patient response to treatment.

Many studies have shown increased marrow fat during OP (42–47). In our study, it was clear that CBWPD was more sensitive to the changes in bone than BMFF, evidenced by the CBWPD’s improved correlations with BMD, T score, and FRAX score over BMFF, as well as its superior performance in terms of cohort discrimination. CBWPD’s increased sensitivity to bone changes is not surprising because BMFF does not reveal true bone loss: some patients with normal BMFF have been reported to have bone loss or abnormal bone mineralization (48).

There are several limitations in this study. First, the scan time of the CBWPD measurement protocol was relatively long (around 12 min in total) compared to clinical sequences. Advanced image reconstruction methods such as compressed sensing or deep learning could be incorporated to accelerate the scan with comparable image quality (49, 50) in the future. Second, we did not follow up on the fracture rate of these patients for 5-10 years, though such a long-term study will be completed in future work. The longitudinal study would be valuable in further validating the proposed technique and in providing information about collagen matrix changes that accompany aging.

In conclusion, the STAIR-UTE-measured CBWPD is a promising biomarker for evaluation of the bone changes in OP and of osteoporotic fracture risk.

## Data Availability Statement

The original contributions presented in the study are included in the article/supplementary material. Further inquiries can be directed to the corresponding author.

## Ethics Statement

The studies involving human participants were reviewed and approved by ethical approval from the institutional review board of The Fifth Affiliated Hospital of Sun Yat-Sen University. The patients/participants provided their written informed consent to participate in this study.

## Author Contributions

JL, S-LL, and Y-JM conceived the study. WL, X-JC, J-XF, LY, P-HH, Z-HS, and HL collected the data. JL, Y-JM, and Y-TL analyzed the data. All the authors collaborated in interpretation of the results and drafting and revision of the report. All authors contributed to the article and approved the submitted version.

## Funding

This work was supported by the National Natural Science Foundation of China (Grant Nos. 82172053).

## Conflict of Interest

Author Y-TL was employed by GE Healthcare.

The remaining authors declare that the research was conducted in the absence of any commercial or financial relationships that could be construed as a potential conflict of interest.

## Publisher’s Note

All claims expressed in this article are solely those of the authors and do not necessarily represent those of their affiliated organizations, or those of the publisher, the editors and the reviewers. Any product that may be evaluated in this article, or claim that may be made by its manufacturer, is not guaranteed or endorsed by the publisher.
